# Type Vb Double Common Bile Duct: A Rare Case Associated With Cholangiocarcinoma

**DOI:** 10.7759/cureus.70118

**Published:** 2024-09-24

**Authors:** Sahil M Patel, Varshini Venkatesan, Kenneth M Sigman, Mohannad F Dugum

**Affiliations:** 1 Internal Medicine, Brookwood Baptist Health, Birmingham, USA; 2 Gastroenterology, Grandview Medical Center, Birmingham, USA

**Keywords:** acute cholangitis, anatomical variation, cholangiocarcinoma, double common bile duct, endoscopic retrograde cholangiopancreatography

## Abstract

Double common bile duct (DCBD), also called extrahepatic biliary duct duplication, is a rare anatomical variation of the biliary anatomy that involves either the presence of a septum within the common bile duct (CBD) or an accessory CBD. The first case of DCBD was reported by Vesarius in 1543. A classification system for DCBD that included five types was proposed in 2007. Type V DCBD involves a duplicated extrahepatic bile duct with common drainage of both ducts into the duodenum and can be further divided into type Va, where there are no communicating channels, and type Vb, where there are one or more communicating channels. By 2021, only eight cases of type V DCBD had been reported, of which only two were type Vb DCBDs. As far as we know, this is the third reported case of type Vb DCBD. In addition to choledocholithiasis, cholangitis, and pancreatitis, DCBD has been associated with an increased risk of malignancies such as cholangiocarcinoma and upper gastrointestinal tract cancers.

Here, we present a case of a 28-year-old female with intrahepatic cholangiocarcinoma undergoing chemotherapy who was referred to our hospital for evaluation of worsening jaundice and suspicion of infected percutaneous transhepatic cholangiography (PTC) drain. After extensive investigation, she was found to have a type Vb DCBD, which meant that her PTC drain was only providing partial therapy for her biliary obstruction. Following the placement of metal stents in both CBDs, her jaundice resolved, allowing her to continue her chemotherapy regimen. In conclusion, this case highlights one of the rarest bile duct anatomical variations, a type Vb DCBD, as well as the importance of evaluating young cholangiocarcinoma patients with magnetic resonance cholangiopancreatography (MRCP) for the presence of a DCBD, especially when they present with worsening jaundice despite receiving appropriate therapy. These patients require stenting of both CBDs to properly address their biliary obstruction.

## Introduction

Double common bile duct (DCBD), also called extrahepatic biliary duct duplication, is a rare variation of biliary anatomy that involves either the presence of a septum within the common bile duct (CBD) or an accessory common bile duct [1]. The first case of DCBD was reported by Vesarius in 1543, and only 24 cases had been reported in Western literature by 1986 [2]. In 2002, Yamashita et al. [3] reported 47 cases in Japanese literature, and in 2014, Chen et al. [4] reported 24 cases in Chinese literature. Goor and Ebert described the earliest classification of DCBD, which included four variations: type I, type II, type III, and type IV [1]. This was later modified by Saito et al. [5] and then Choi et al. [6], who classified a new variation of DCBD called type V, which became the currently recognized classification system. Type V DCBD involves a duplicated extrahepatic bile duct with common drainage of both ducts into the duodenum [7]. Type V is then further subdivided into type Va, where there are no communicating channels, and type Vb, where there are one or more communicating channels [7]. We discuss the different variations in more detail in the Discussion section. By 2021, only eight cases of type V DCBD had been reported, of which only two were type Vb DCBD [2]. As far as we know, this is the third reported case of type Vb DCBD. In addition to choledocholithiasis, cholangitis, and pancreatitis, DCBD has been associated with an increased risk of malignancies such as cholangiocarcinoma and upper gastrointestinal tract cancers with Yamashita et al. reporting malignancies in 25.5% of reported DCBD cases [3]. In this report, we present the case of a young female with cholangiocarcinoma who was found to have a type Vb DCBD only after she developed cholangitis from incomplete treatment of her biliary obstruction.

This article was previously presented as a meeting abstract at the 2023 American College of Gastroenterology Annual Meeting on October 22, 2023.

## Case presentation

A 28-year-old female with a past medical history of cholecystectomy was diagnosed with intrahepatic cholangiocarcinoma after developing painless jaundice at an outside hospital. She had no family history of any malignancies. She was referred to oncology and started on chemotherapy. Six months later, she had a percutaneous transhepatic cholangiography (PTC) drain placed for an increase in her serum bilirubin at an outside hospital. She subsequently developed encephalopathy, dyspnea, and abdominal pain two days after PTC drain placement. She was then transferred to our center for management of a presumably infected PTC drain. Upon arrival, her vital signs were significant for tachycardia at 133 beats/minute and tachypnea at 22 breaths/minute. Laboratory work was significant for a leukocyte count of 11×10^9^/L, total bilirubin of 2.6 mg/dL, direct bilirubin of 1.6 mg/dL, alanine aminotransferase (ALT) of 33 U/L, aspartate aminotransferase (AST) of 44 U/L, and alkaline phosphatase (ALP) of 134 U/L (Table 1). The patient was started on broad-spectrum antibiotics for sepsis secondary to acute cholangitis.

**Table 1:** Laboratory results WBC, white blood cells; ALT, alanine aminotransferase; AST, aspartate aminotransferase; ALP, alkaline phosphatase

Laboratory test	Result	Reference range
WBC	11×10^9^/L	4-10×10^9^/L
Total bilirubin	2.6 mg/dL	0.2-1 mg/dL
Direct bilirubin	1.6 mg/dL	0-0.2 mg/dL
ALT	33 U/L	12-78 U/L
AST	44 U/L	15-37 U/L
ALP	134 U/L	45-117 U/L

**Figure 1:**
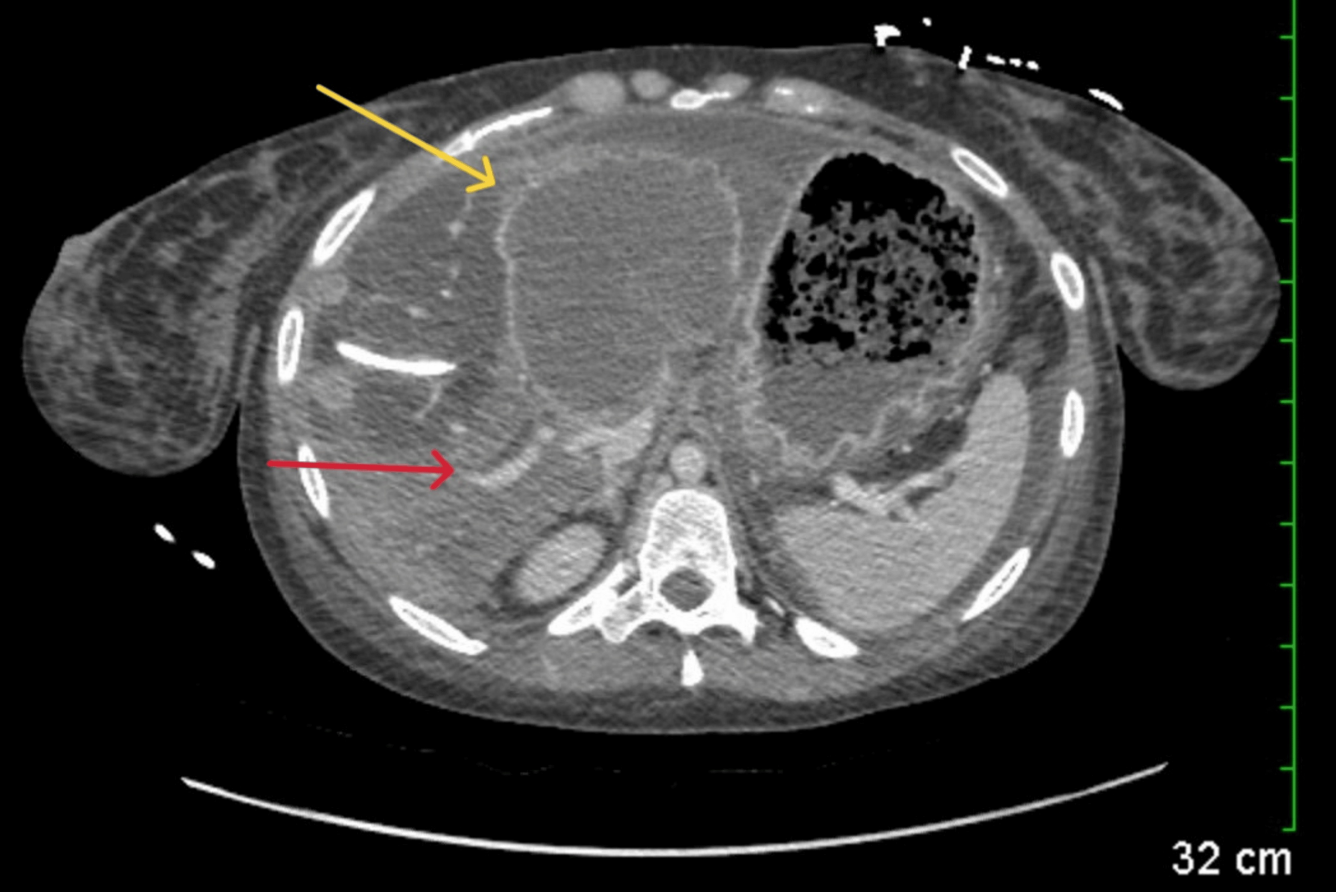
Abdominal CT with IV contrast showing multiple peripherally enhancing masses within the liver (yellow arrow), as well as bilateral intrahepatic biliary ductal dilation (red arrow) CT, computed tomography; IV, intravenous

**Figure 2:**
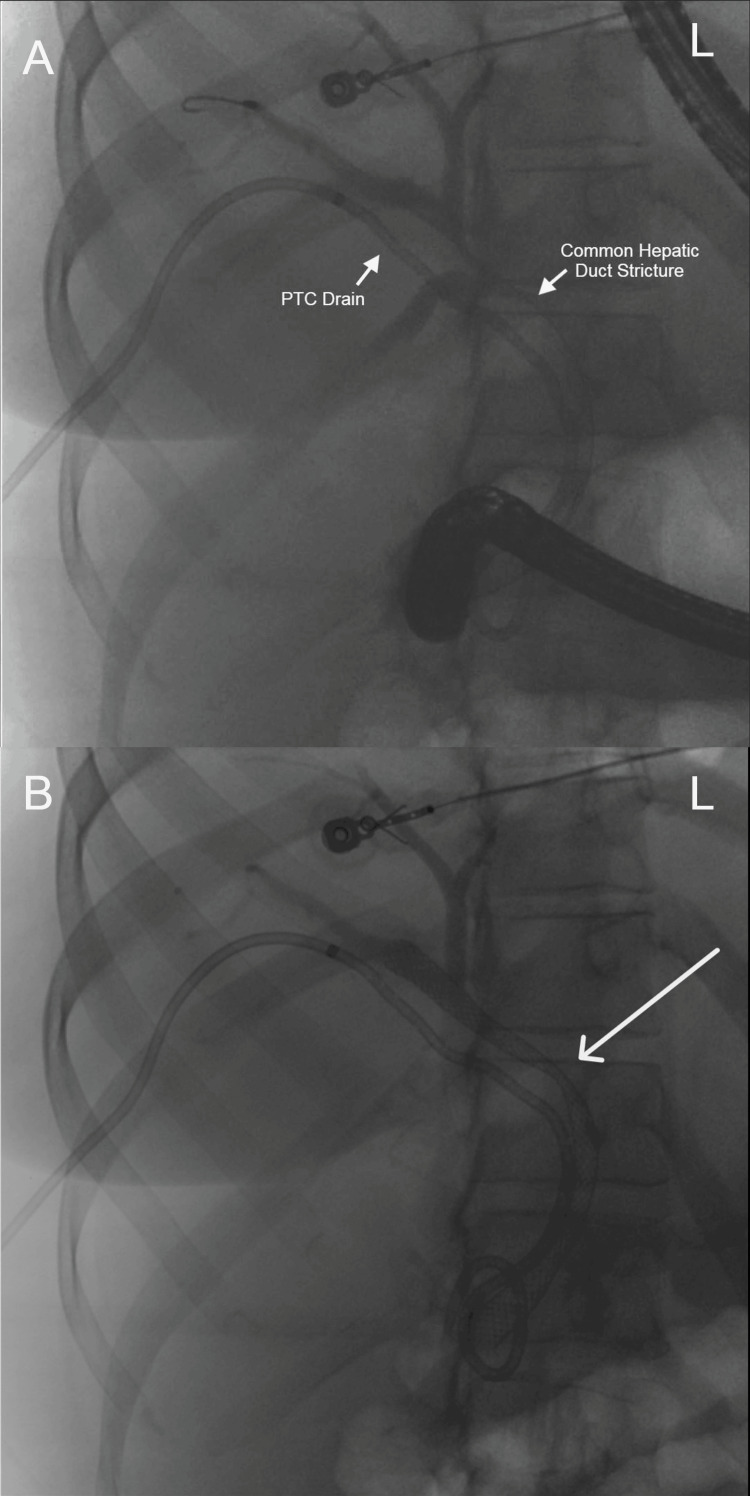
Panel A shows a cholangiogram showing normal CBD with a mild stricture in the common hepatic duct, with the PTC drain in an entirely separate ductal system, and panel B shows the metal stent placed in the CBD, extending from the common hepatic duct to the major papilla (white arrow) CBD, common bile duct; PTC, percutaneous transhepatic cholangiography

**Figure 3:**
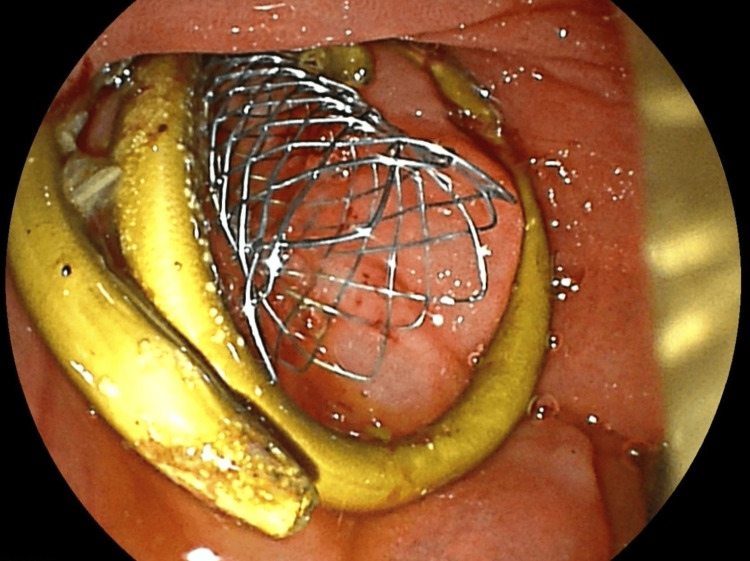
Endoscopic image of the metal stent and PTC drain emerging from the major papilla adjacent to one another PTC, percutaneous transhepatic cholangiography

**Figure 4:**
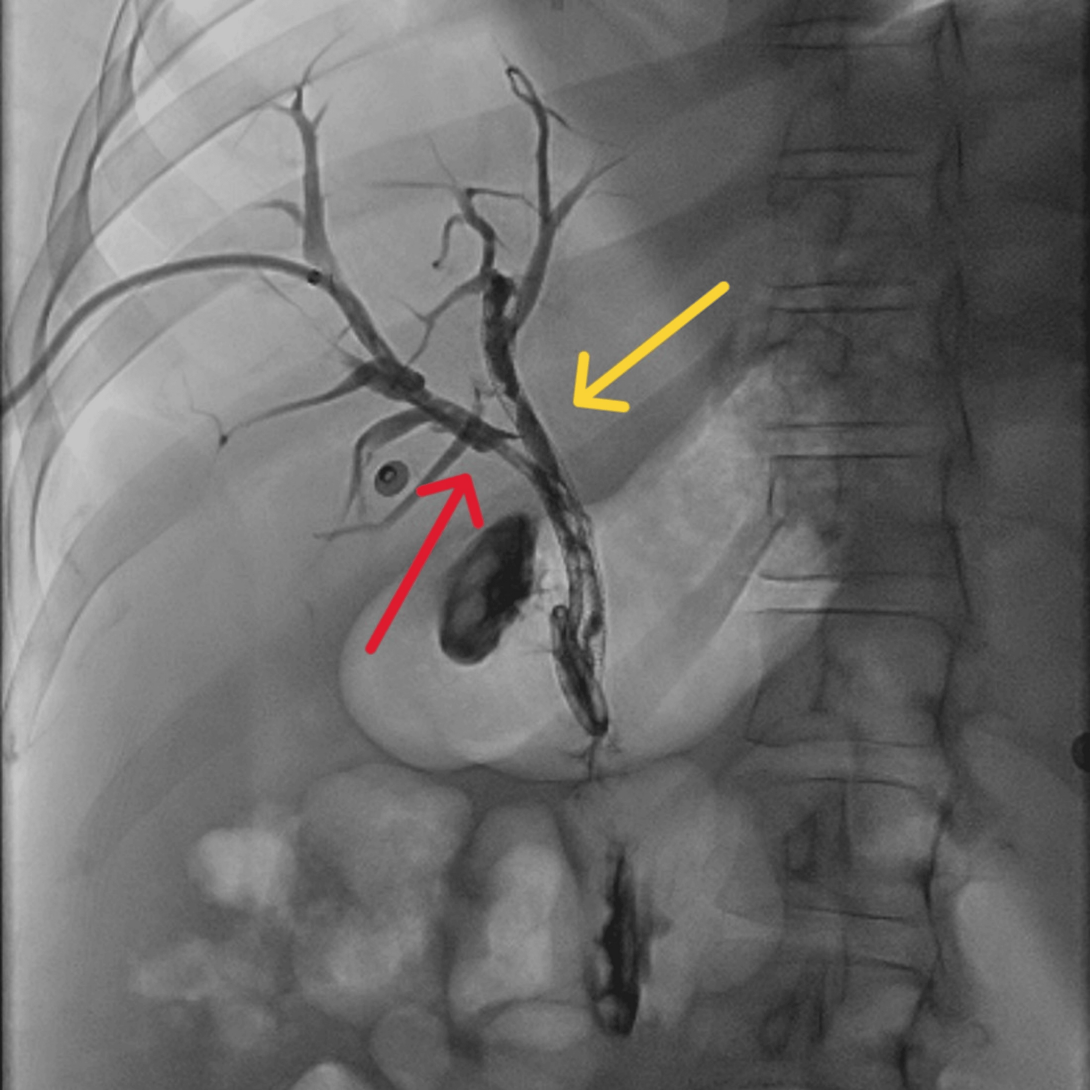
Cholangiogram performed through the PTC drain showing the presence of a DCBD (the PTC drain can be seen in the right CBD (red arrow) and the metal stent in the left CBD (yellow arrow)) PTC, percutaneous transhepatic cholangiography; DCBD, double common bile duct; CBD, common bile duct

**Figure 5:**
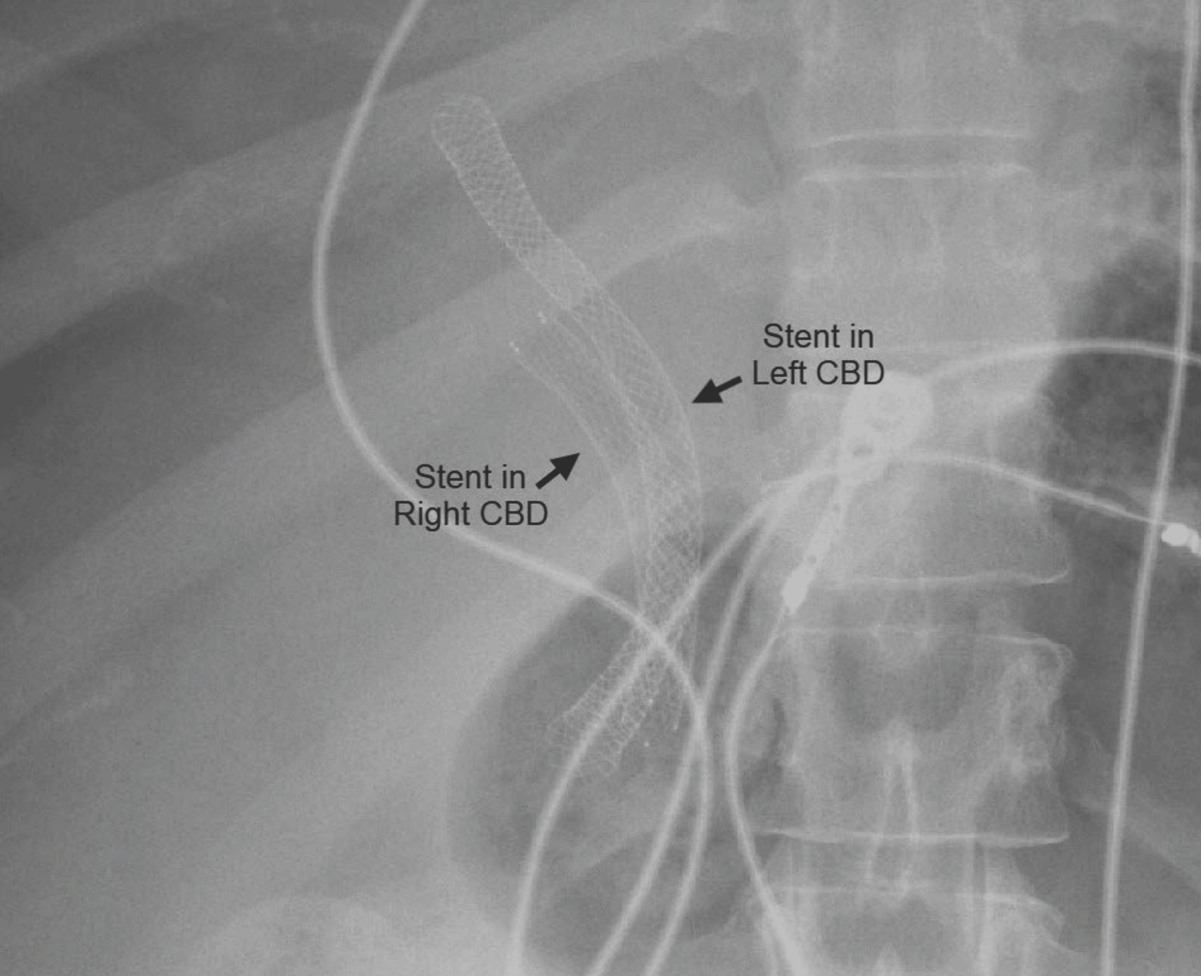
Abdominal radiograph showing the presence of the endoscopically placed metal stent in the left CBD and the IR-placed metal stent in the right CBD (both metal stents can be seen exiting the biliary system through the major papilla) CBD, common bile duct; IR, interventional radiology

Abdominal computed tomography (CT) with IV contrast showed multiple peripherally enhancing masses within the liver, as well as bilateral intrahepatic biliary ductal dilation (Figure 1). Endoscopic ultrasound revealed multiple large solid masses in the liver consistent with metastases and excluded a fluid component, i.e., abscess. Due to the acute cholangitis, endoscopic retrograde cholangiopancreatography (ERCP) was performed, and the PTC drain was visualized emerging from the major papilla. The common bile duct was then cannulated using a balloon adjacent to the PTC drain, and contrast was injected. The cholangiogram showed a normal CBD and a mild stricture in the common hepatic duct, but the PTC drain was in an entirely separate ductal system (Figure 2A). A metal stent was then placed into the CBD and extended from the common hepatic duct to the major papilla (Figure 2B). The metal stent emerged from the major papilla adjacent to the PTC drain (Figure 3). Due to concern about the misplacement of the PTC drain, the interventional radiology (IR) team interrogated the drain using contrast (Figure 4). This revealed the presence of a DCBD, with the PTC drain placed in the right CBD and an endoscopically placed metal stent in the left CBD. Consecutive images from the contrast study showed a single proximal communication between the two CBDs. The IR team then removed the PTC drain, and a metal stent was placed in the right CBD, with both stents joining at the major papilla, thus confirming the presence of type Vb DCBD (Figure 5). Following the placement of both metal stents, the patient's serum bilirubin levels normalized, and symptoms improved to where she was safely discharged home to continue her palliative chemotherapy regimen with her oncologist. Upon following up with her oncologist, her serum bilirubin was 1 mg/dL. The patient ultimately elected to pursue home hospice care shortly afterward.

## Discussion

During early embryogenesis, the presence of a DCBD is normal [7]. However, this quickly regresses to give rise to the conventional biliary anatomy with a single CBD emptying bile into the duodenum [7]. Therefore, the development of a DCBD is believed to be due to disruption in early embryogenesis and preservation of the extrahepatic accessory duct [6]. As mentioned earlier, DCDB is a rare congenital anomaly, with type Vb DCBD being exceedingly rarer with only two reported cases [2]. Unfortunately, the true prevalence of DCBD may never be known because most individuals are asymptomatic.

**Figure 6:**
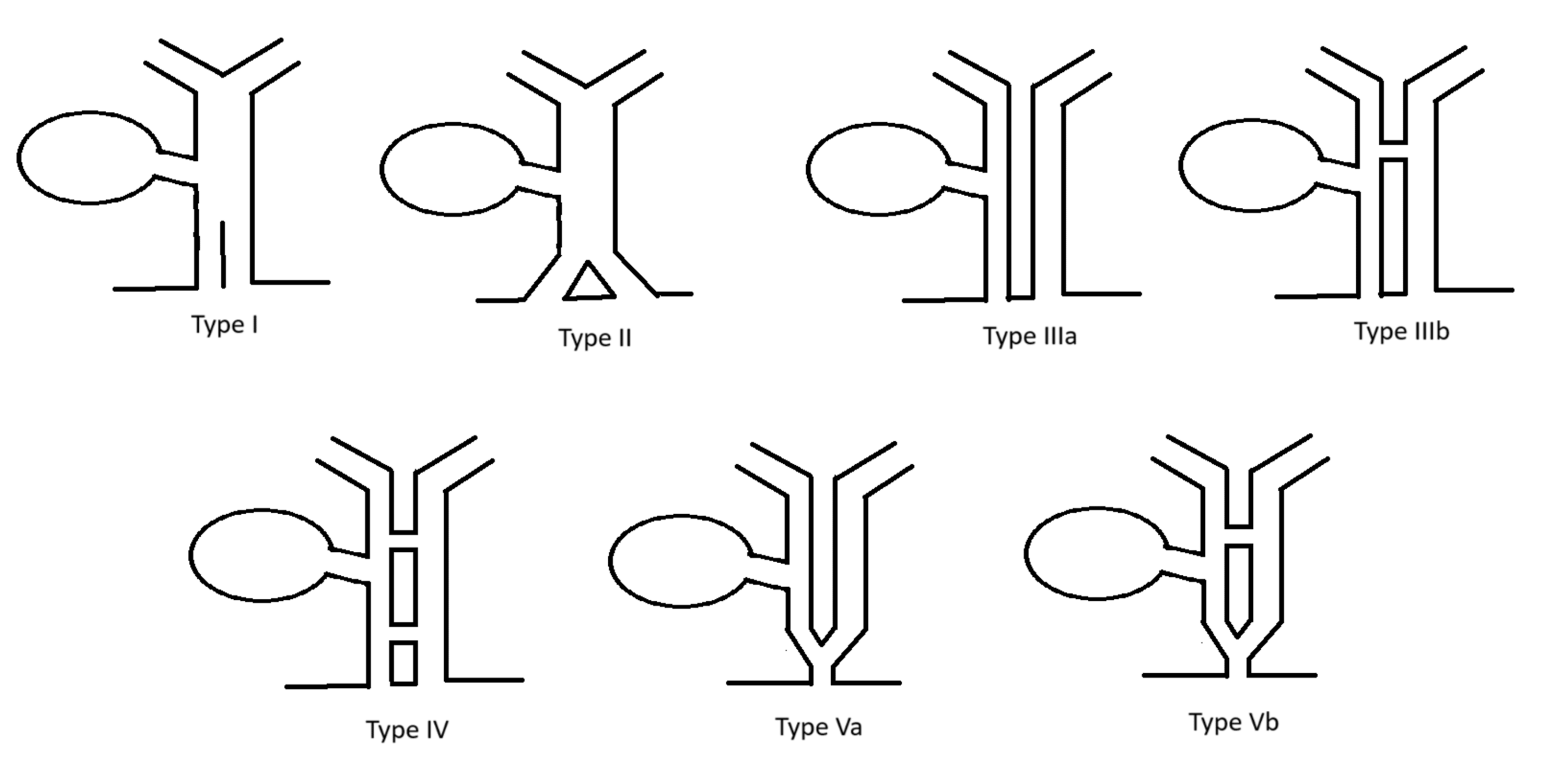
Classification of double common bile duct Modified double common bile duct classification proposed by Choi et al. [6]: type 1 with a spectrum dividing the common bile duct; type II with a distal bifurcation of the common bile duct with independent drainage into the duodenum; type III with duplicated extrahepatic bile ducts with independent drainage into the duodenum without (a) or with (b) intrahepatic communicating channels; type IV with duplicated extrahepatic bile ducts and extrahepatic communicating channels with or without intrahepatic communicating channels; and type V with duplicated extrahepatic bile ducts with common drainage of both ducts into the duodenum either without (a) or with (b) communicating channels. Our case describes a patient with type Vb DCBD. DCBD, double common bile duct

The currently recognized classification of DCBD was proposed by Choi et al. [6] and is an adaptation from the prior classification system by Saito et al. [5]. This classification system includes five types (Figure 6) [6]. Type I is characterized by a septum dividing the CBD [7]. Type II is characterized by a distal bifurcation of the CBD with independent drainage into the duodenum [7]. Type III involves duplicated extrahepatic bile ducts with independent drainage into the duodenum [7]. This is further divided into type IIIa, where there are no intrahepatic communicating channels, and type IIIb, where there is at least one intrahepatic communicating channel [7]. Type IV is characterized by duplicated extrahepatic bile ducts with independent drainage into the bowel with either extrahepatic communicating channels or both extrahepatic and intrahepatic communicating channels [7]. Type V involves duplicated extrahepatic bile ducts with common drainage of both ducts into the duodenum [7]. This is also further divided into type Va, where there are no communicating channels, and type Vb, where there are one or more communicating channels [7].

As evidenced by Figure 5, our patient had a type Vb DCBD as there were two separate extrahepatic bile ducts with a single proximal communicating channel that both had common drainage into the duodenum. As mentioned earlier, there are only two other cases of type Vb DCBD reported thus far [8,9]. Both cases involved older female patients, 81-years-old and 44-years-old, respectively, who both presented with cholangitis due to choledocholithiasis [8,9], unlike our case where she presented with cholangitis due to cholangiocarcinoma causing biliary obstruction.

DCBD has been associated with an increased risk of choledocholithiasis, cholangitis, pancreatitis, choledochal cysts, and malignancies such as cholangiocarcinoma and upper gastrointestinal tract cancers [3]. Therefore, most new cases of DCBD are found incidentally during the management of other conditions. As the management of these conditions frequently involves endoscopic and/or surgical procedures, correct identification of DCBD is crucial. Hoepfner et al. detailed the increased risk of surgical complications when DCBD is not properly identified during the initial intervention [10]. When DCBD is suspected, either ERCP or magnetic resonance cholangiopancreatography (MRCP) is recommended [9]. However, since ERCP is more invasive and has a higher complication risk, MRCP is the preferred diagnostic modality [9].

Management varies depending on the type of DCBD. In type I, resection of the septum dividing the CBD is recommended [9]. In types II, III, and IV, patients should have the accessory bile duct resected, especially if it opens into the pancreas or stomach [9]. Given the few cases of type V, there is no clear strategy on how to manage them, so as a result, strict monitoring is recommended [9].

In our case, the patient had early-onset cholangiocarcinoma with a type Vb DCBD, which was not identified until six months after initial diagnosis. Unfortunately, not identifying the DCBD during the initial evaluation led to only partial therapy of her biliary obstruction, ultimately causing acute cholangitis. This warranted an ERCP, which is the gold standard, and the eventual placement of metal stents in both CBDs, thus appropriately relieving the biliary obstruction.

## Conclusions

This case highlights one of the rarest bile duct anatomical variations, a type Vb DCBD, as well as the importance of evaluating young cholangiocarcinoma patients with MRCP for the presence of a DCBD, especially when they present with worsening jaundice despite receiving appropriate therapy. These patients require stenting of both CBDs to properly address their biliary obstruction. Failing to properly identify DCBD in these patients allows for biliary obstructions to persist despite the placement of a single biliary stent. This allows for worsening of their jaundice and the development of acute cholangitis.
